# Dynamic motions of ice-binding proteins in living *Caenorhabditis elegans* using diffracted X-ray blinking and tracking

**DOI:** 10.1016/j.bbrep.2022.101224

**Published:** 2022-02-03

**Authors:** Masahiro Kuramochi, Yige Dong, Yue Yang, Tatsuya Arai, Rio Okada, Yoichi Shinkai, Motomichi Doi, Kouki Aoyama, Hiroshi Sekiguchi, Kazuhiro Mio, Sakae Tsuda, Yuji C. Sasaki

**Affiliations:** a Graduate School of Science and Engineering, Ibaraki University, Hitachi, 316-8511, Japan; b Graduate School of Frontier Sciences, The University of Tokyo, Kashiwa, 277-8561, Japan; c AIST-UTokyo Advanced Operando-Measurement Technology Open Innovation Laboratory (OPERANDO-OIL), National Institute of Advanced Industrial Science and Technology (AIST), Kashiwa, 277-8565, Japan; d Biomedical Research Institute, National Institute of Advanced Industrial Science and Technology (AIST), Japan; e Center for Synchrotron Radiation Research, Japan Synchrotron Radiation Research Institute, 1-1-1, Kouto, Sayo-cho, Sayo-gun, Hyogo, 679-5198, Japan

**Keywords:** Diffracted X-ray tracking (DXT), Diffracted X-ray blinking (DXB), C. elegans, Ice-binding protein

## Abstract

The dynamic properties of protein molecules are involved in the relationship between their structure and function. Time-resolved X-ray observation enables capturing the structures of biomolecules with picometre-scale precision. However, this technique has yet to be implemented in living animals. Here, we examined diffracted X-ray blinking (DXB) and diffracted X-ray tracking (DXT) to observe the dynamics of a protein located on intestinal cells in adult *Caenorhabditis elegans*. This *in vivo* tissue-specific DXB was examined at temperatures from 20 °C to −10 °C for a recombinant ice-binding protein from *Antarctomyces psychrotrophicus* (AnpIBP) connected with the cells through a transmembrane CD4 protein equipped with a glycine-serine linker. AnpIBP inhibits ice growth at subzero temperatures by binding to ice crystals. We found that the rotational motion of AnpIBP decreases at −10 °C. In contrast, the motion of the AnpIBP mutant, which has a defective ice-binding ability, did not decrease at −10 °C. The twisting and tilting motional speeds of AnpIBPs measured above 5 °C by DXT were always higher than those of the defective AnpIBP mutant. These results suggest that wild-type AnpIBP is highly mobile in solution, and it is halted at subzero temperatures through ice binding. DXB and DXT allow for exploring protein behaviour in live animals with subnano resolution precision.

## Highlights

• Dynamic motions of ice-binding protein (AnpIBP) were analysed in live animals using *in vivo* DXB and DXT techniques.
• The rotational motion of AnpIBP decreases at −10 °C, whereas that of the defective AnpIBP mutant did not decrease at −10 °C.
• The twisting and tilting motions of AnpIBPs measured above 5 °C were always higher than those of the defective AnpIBP mutant.

## 1 Introduction

The dynamic properties of protein molecules are important for understanding their function related to their structure. The dynamic motions of molecules *in vivo* can be accessed by fluorescent imaging, but the length scales probed in fluorescent imaging typically exceed several tens of nanometres because of the large wavelength of visible light. Time-resolved X-ray diffraction techniques have the unique potential to measure the dynamic motion of biomolecules at the picometre length scale. Diffracted X-ray tracking (DXT) uses white- or pink-beam X-rays to track X-ray diffraction spots from protein-labelled gold nanocrystals. DXT can detect tilting and twisting motions of protein molecules with angular velocities in the θ and χ directions with milli-radian accuracy [1] and has been used to analyse several biomolecules, such as the major histocompatibility complex (MHC) class II protein, acetylcholine-binding protein (AChBP), nicotinic acetylcholine receptor (nAChR), and TRPV1 channel [[2], [3], [4], [5]]. However, DXT requires broadband X-rays at synchrotron radiation facilities to track the diffraction spots from gold nanocrystals. Because such broadband width X-rays with high flux physically and chemically influence biomolecules via strong radiation damage, observations over long time scales have restrictions.

Recently, we developed a DXT-like technique by using a monochromatic X-ray beam, which we term “Diffracted X-ray blinking” (DXB) [6]. In DXB, the diffraction spots are observed to cycle in and out of the Bragg condition, and they appear as blinking. Blinking includes tilting and twisting motions of biomolecules and can be evaluated as autocorrelation-function (ACF) exponential relaxation. This ACF analysis cannot separately evaluate the tilting and twisting motions, but DXB provides several benefits for X-ray biomolecular observation. DXB can observe molecular motion on a long time scale with a low X-ray dose and can also be performed with a laboratory X-ray source, not just with synchrotron radiation facilities. DXT and DXB have been used to observe the dynamic motions of protein molecules at the atomic scale by using X-rays and nanocrystal probes, but they have yet to be implemented in living animals.

The nematode *Caenorhabditis elegans* is a useful model organism for understanding unsolved complex biological questions, such as nervous system function and developmental properties. In addition, *C. elegans* has also been used to establish many biological methods, such as fluorescent imaging, RNAi, and genome-editing techniques [[7], [8], [9]]. It is suitable as the first animal to demonstrate cutting-edge X-ray measurements for single molecular observations *in vivo*. In addition, *C. elegans* also shows tolerance to low linear energy-transfer (LET) radiation, such as X-rays and γ-rays [[10], [11], [12]]. These characteristics are powerful advantages for the observation of biomolecular motions with DXT and DXB. These X-ray diffraction techniques should be capable of monitoring tilting and twisting motions at the atomic scale of single molecules in living worms.

In this paper, we focused on ice-binding protein (IBP) from *Antarctomyces psychrotrophicus* (AnpIBP) [13]. AnpIBP inhibits ice growth by binding to ice crystals through its ice-binding site. The AnpIBP mutant, in which Thr156 in the AnpIBP ice-binding site was mutated to a tyrosine residue, has a reduced ice-binding ability [14]. Our previous paper reported that AnpIBP improves the freezing tolerance of *C. elegans*. The defective AnpIBP mutant did not improve its tolerance because the mutation reduced its ice-binding ability [15]. The freezing tolerance of *C. elegans* was correlated with the ice-binding ability of IBPs. These results suggest that AnpIBP inhibits ice crystal growth in living worm cells and protects against cell injury by ice crystals. However, it is not clear how the wild-type AnpIBP and the AnpIBP mutant interact with ice crystals *in vivo*.

Atomistic molecular dynamics simulations support detailed structural and dynamical aspects of ice-binding ability. IBP binds to ice crystals and is then fixed on the ice surface, while the defective IBP mutant is unable to bind and diffuses ahead of the growing ice front [16,17]. Based on those simulations, the wild-type and the mutant IBPs should show different behaviour towards ice crystals. If so, those different behaviours can be detected as the different rotational diffusion of IBPs by DXB and DXT.

Here, we measured the dynamic motion of AnpIBPs in live *C. elegans*. To express AnpIBPs to the cell surface, the transmembrane protein CD4 was used, and gold nanocrystals were attached via a thiol site of CD4. Rotational motion of transmembrane CD4 and ice-binding proteins was observed with picometre precision by DXB. Interestingly, the rotational motion of AnpIBP significantly decreased at −10 °C, whereas that of the AnpIBP mutant did not decrease. These motions are related to their ice-binding ability. In addition, the twisting and tilting motions of AnpIBPs measured by DXT have precisions of approximately 0.4–1.0 mrad and 0.9–2.6 mrad, respectively. Interestingly, the angular speeds for the twisting and tilting motion of AnpIBPs measured above 5 °C were always higher than those of the defective AnpIBP mutant. The rotational motions of AnpIBPs can be quantitatively monitored at the picometre or milliradian level in live animals using DXB and DXT. DXB and DXT are expected to open the possibility to explore the molecular basis of complex biological phenomena such as pathological cellular processes and neural circuit regulation with atomic-scale precision directly in live animals.

## 2 Materials and methods

### 2.1 Molecular biology

Standard methods were used to construct the plasmids. The plasmid expressing the CD4 cDNA optimized for *C. elegans* was a gift from E. Feinberg. The human T cell protein CD4 acts as an extracellular ligand of the MHC class II protein [18]. *C. elegans* does not express the CD4 protein, but CD4 has been used in bioimaging techniques for molecular observations of the plasma membrane in *C. elegans* [19,20]. To express AnpIBP and a transmembrane CD4 complex, we fused CD4 to the C-terminus of AnpIBP through a glycine-serine linker (GGGGSGGGGS). This coding sequence was inserted between the *Nhe*I and *Sal*I sites of the pPD95.79 vector. Then, the promoter region for cell-specific expression of the cDNAs was inserted between the *Not*I and *BamH*I sites of the resulting pPD95.79/AnpIBP:CD4 plasmid. We used the *elt-2* promoter for intestinal cell-specific expression.

### 2.2 Strains

To generate transgenic animals, plasmid DNA was injected into *lin-15* mutant animals using a standard microinjection method [21]. We used the following strains: wild-type *C. elegans* variety Bristol strain (N2), CMS21 *lin-15 (n7*65ts*); Ex[elt-2p∷CD4]*, CMS22 *lin-15 (n7*65ts*); Ex[elt-2p:AnpIBP:CD4]*, CMS23 *lin-15 (n7*65ts*); Ex[elt-2p:AnpIBP T156Y:CD4]*.

Worms were cultivated on standard nematode growth medium (NGM) plates seeded with *E. coli* OP50 at room temperature (∼24 °C).

### 2.3 Gold nanocrystal labelling of cell membranes in *C. elegans*

Gold nanocrystals were fabricated via epitaxial growth on cleaved KCl (100) under a 10^−4^ Pa vacuum. The size of the gold nanocrystals is approximately 60–80 nm. The nanocrystals were mixed with phosphate-buffered saline (PBS) and sonicated at approximately 0 °C for 1 h. Adult worms were incubated in PBS with gold nanocrystals for 4 h at room temperature. Transmembrane CD4 is located on intestinal cells. The worms swallow gold nanocrystals during 4 h of incubation. Their intestinal cells contact the gold nanocrystals, and then the gold nanocrystals bind to a thiol site on CD4. After incubation with the gold, the worms were washed with PBS and placed in the sample holder.

### 2.4 Diffracted X-ray blinking (DXB)

DXB was performed using a laboratory X-ray source (Rigaku FR-D: Cu anode, 50 kV, 60 mA). Time-resolved diffraction images were recorded using a 2D photon-counting detector (Pilatus 100K-A, Dectris, Switzerland). The sample temperature was controlled by a temperature-controlled microscope stage (10,084 L, Linkam Scientific Instruments, UK).

The time-resolved diffraction intensity in Au(111) was analysed at each pixel by the autocorrelation function (ACF),

$$I\left(\tau \right)=\frac{<I\left(t\right)I\left(t+\tau \right)>}{<I{\left(t\right)}^{2}>}$$

where I(t) is the diffraction intensity, the brackets < > indicate the time-averaged value, and τ is the lag time (or interval). The ACF can be computed by changing τ and then fitted to an exponential curve by ACF(t) = Aexp(−Γt) + y, where A is the amplitude, y is the conversion factor, and Γ is the decay constant. We chose decay constants to satisfy the following conditions: (I) 0 < y, 0 < A and 0 < Г, and (II) residual values between the fitted and actual ACF curves of less than 1.0 [[22], [23], [24]]. These calculations were performed for all pixels. The distribution of the decay constants of the Au(111) diffraction ring was visualized using histograms and box plots to estimate the dynamic behaviour of the protein molecules.

The rotational diffusion coefficient D_R_ of a single colloidal sphere with radius r is defined by the Stokes–Einstein–Debye relationship [[25], [26], [27]]. The ACF decay constant Г is related to the rotational diffusion coefficient of single grains D_R_, as expressed in the following equation:

$$\begin{array}{l} D_{R}=\frac{{\varphi_{\theta }}^{2}\text{Γ}}{4} \end{array}$$

The rotational displacement φ_θ_ was calculated by the angular width of the 2θ diagram.

### 2.5 Diffracted X-ray tracking (DXT)

DXT was performed using the SPring-8 BL40XU beamline. The beam size was adjusted to 50 μm in diameter by inserting a pinhole aperture upstream of the sample, and time-resolved diffraction images from the gold nanocrystals were recorded by an X-ray image intensifier (V7739P, Hamamatsu photonics) and a CMOS camera (ORCA FLASH 4.0, Hamamatsu photonics). For each sample, diffractions at several positions (approximately 10 trials) were manually recorded. The distance between the sample and the detector was set to 50 mm. X-rays with an energy bandwidth of 0.1 (15.8 keV in peak energy and photon flux of 10^11^ photons/s) were used for the living-worm DXT. Images were captured at a rate of 10 msec/frame. Diffraction spots were tracked by TrackPy (v0.3.2 https://doi.org/10.5281/zenodo.60,550), and the trajectories were analysed using custom software written within IGOR Pro (Wavemetrics, Lake Oswego). The sample temperature was controlled by a custom-made heating and cooling stage.

Hundreds of diffraction spots from Au(111) and Au(200) in each trial were tracked and were assigned as θ and χ directional movements. Angular displacement histograms with each temperature condition were fitted by the Gaussian distribution.

## 3 Results

### 3.1 Labelling of gold nanocrystals for X-ray observation of molecular motion in *C. elegans*

**Fig. 1:**
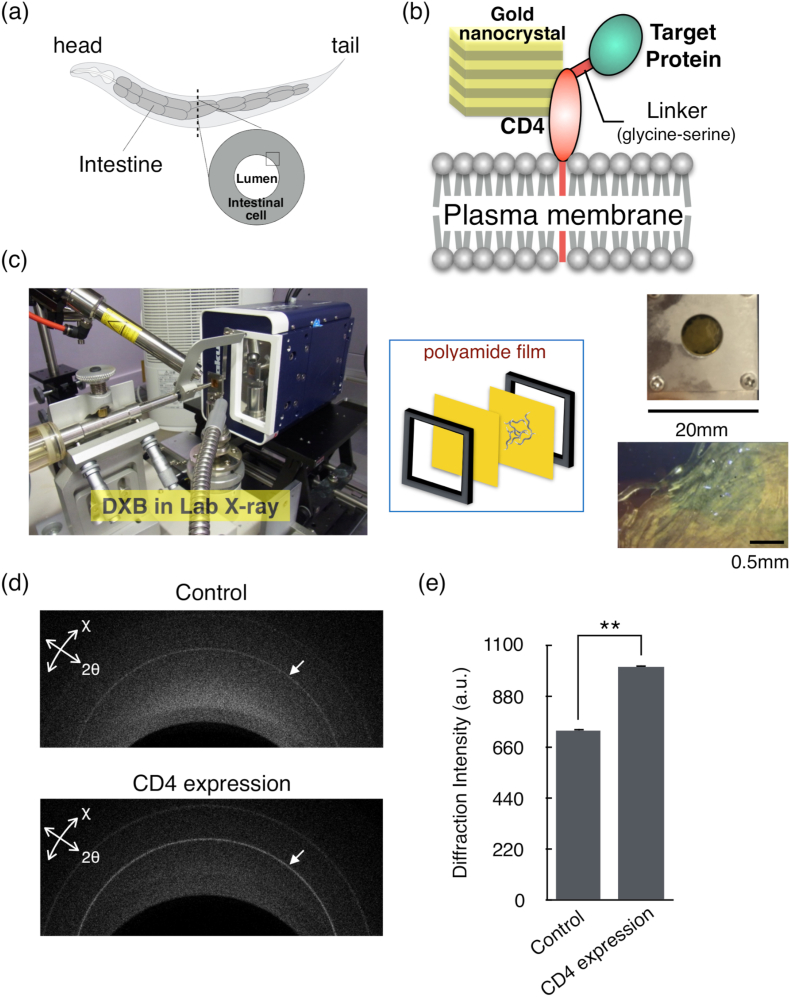
Labelling of gold nanocrystals for X-ray molecular observation in *C. elegans*. (a) Intestine within *C. elegans*. (b) Schematic illustration of gold nanocrystal labelling for CD4 and the target protein complex on the intestinal cell membrane. Gold nanocrystals are labelled through thiol sites on CD4. (c) DXB measurement system with a cooling stage. Hundreds of worms were placed between polyamide films and set in a sample holder. (d) X-ray diffraction images. The images were integrated from the 2000 images with 200 ms exposure. The arrows represent the Au(111) diffraction ring. (e) Diffraction intensity of Au(111) in each sample. Wild-type N2 animals were used as the control data. N = 5. Student's t-test was performed to compare the CD4-expressing worms and wild-type animals. **p < 0.01. (For interpretation of the references to colour in this figure legend, the reader is referred to the Web version of this article.)

To develop DXB and DXT techniques for single molecular observation of living animals, we first constructed a labelling method for protein molecules on the intestinal cell membrane in *C. elegans* by using gold nanocrystals (Fig. 1a and b). For DXB and DXT *in vivo*, gold nanocrystals need to physically bind to target proteins. Here, we focused on the human T cell protein CD4. The transmembrane CD4 is a structurally characterized protein that acts as an extracellular ligand of the MHC class II protein [18]. In our study, by expressing target proteins and the CD4 complex, target proteins on the extracellular side were labelled with gold nanocrystals through CD4. Transgenic worms expressing CD4 on the cell membrane in their intestine were incubated in PBS with gold nanocrystals for 4 h and then placed in the sample holder (Fig. 1c). It is reasonable that the target proteins are located on the plasma membranes of the intestinal cells. Because *C. elegans* swallows gold nanocrystals during feeding, their intestinal cells come into contact with the gold nanocrystals. The thiol site of CD4 on the cell membrane can attach gold nanocrystals [19,28,29] (Fig. 1b). After 4 h of incubation, we rinsed the worms and immediately measured hundreds of worms with a laboratory XRD source. The XRD intensity of the transgenic worms expressing CD4 proteins specifically in intestinal cells was significantly higher than that of wild-type worms (Fig. 1d and e). These results suggest that the CD4 proteins specifically bound to the gold nanocrystals in the intestinal cells.

### 3.2 Dynamic motion of ice-binding proteins in living worms by DXB

**Fig. 2:**
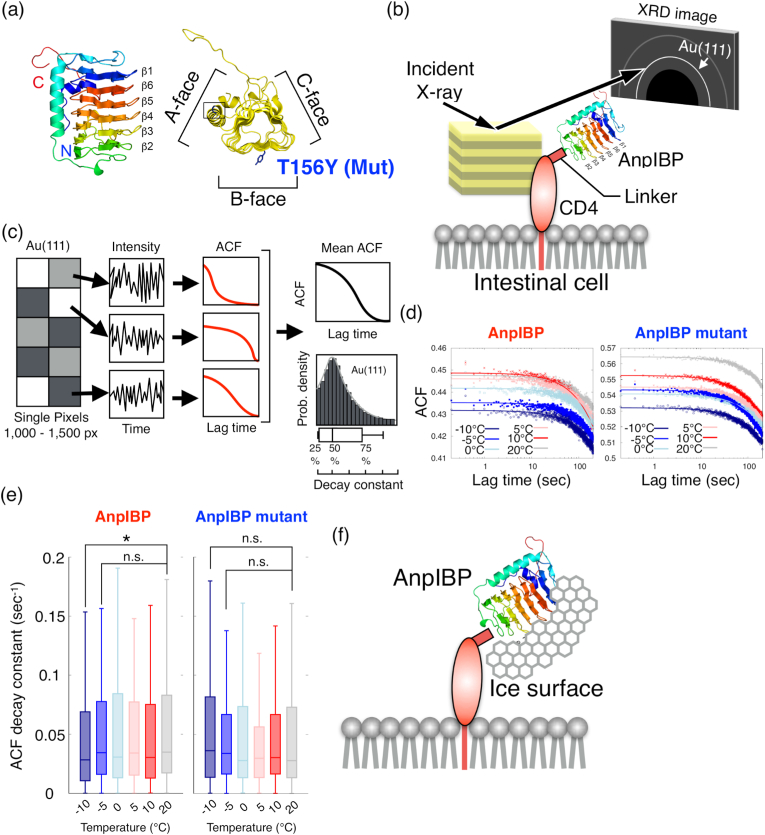
Diffracted X-ray blinking (DXB) for AnpIBPs motion in *C. elegans*. (a) Structure of AnpIBP and the T156Y mutant. (b) Schematic illustration of DXB for observation of the CD4:AnpIBP complex on intestinal cells in live worms. (c) Single pixel (sp) autocorrelation function (ACF) analysis. The time-resolved intensity of single pixels is analysed by sp-ACF. (d) Mean ACF curves in AnpIBP and a defective AnpIBP mutant in *C. elegans*. (e) Boxplot of the ACF decay constant. The boxes show the median and first and third quartiles. The nonparametric Brunner–Munzel test was performed to compare the two samples. *p < 0.05. n.s. represents no significance. (f) Schematic illustration of the AnpIBP-ice complex.

To evaluate the dynamic motion of protein molecules in living worms by our X-ray diffraction techniques, we focused on ice-binding protein from *Antarctomyces psychrotrophicus* (AnpIBP) [13]. AnpIBP consists of three parallel β-sheets (A-face, B-face and C-face) with a triangular cross section, and it inhibits ice crystal growth by binding ice crystals through the B-face (Fig. 2a) [14]. We previously reported that AnpIBP improves the freezing tolerance of *C. elegans*. On the other hand, a defective AnpIBP mutant, which has a T156Y mutation in the B-face, did not improve freezing tolerance because this mutation induces a reduction in ice-binding ability [15]. AnpIBP might inhibit ice crystal growth in living worms and protect against cell injury induced by ice crystals. However, the behaviour of AnpIBP, which binds to ice crystals *in vivo,* has not yet been directly observed. Comparisons between the wild-type and the mutant are useful to understand its structure, dynamics and function. Low X-ray-dose laboratory-DXB can monitor the motion of AnpIBP in living worms over a long time scale.

Here, we measured the dynamic motion of AnpIBP and the CD4 complex in living worms by DXB (Fig. 2b). Blinking of X-ray diffraction from protein-labelled gold nanocrystals includes tilting and twisting motions of biomolecules and can be evaluated as autocorrelation-function (ACF) exponential relaxation. The time-resolved diffraction intensity in Au(111) was analysed at each pixel by a single-pixel ACF (Fig. 2c) [6]. The ACF decay constant relates to the tilting and twisting motion of protein molecules. The ACFs of the wild-type AnpIBP and AnpIBP mutants decayed gradually under each temperature condition (Fig. 2d), implying that AnpIBPs rotate with tilting. Interestingly, the ACF decay constant in AnpIBP was significantly decreased when the temperature decreased to −10 °C from 20 °C (Figs. 2e and S1). The wild-type AnpIBP might generate the AnpIBP-ice complex by binding to ice crystals at subzero temperatures (Fig. 2f). The rotational motion of the AnpIBP-ice complex is probably slower than that of AnpIBP. In contrast, the ACF decay constant of the AnpIBP mutant was not significantly decreased (Figs. 2e and S1). A mutation in the ice-binding site of the AnpIBP mutant causes a weak ice-binding ability [15]. Because the AnpIBP mutant could not bind to ice crystals, the rotational motion of the AnpIBP mutant might not be restricted at −10 °C. Actually, an ice docking simulation has been previously reported in which IBP adsorbs and fixes to the ice surface, while the IBP mutant is unable to bind to the ice and diffuses ahead of the growing ice front [16]. Consistent with the simulation, different behaviours between the wild-type AnpIBP and the AnpIBP mutant under subzero temperature could be observed in the DXB experiments. The rotational diffusion coefficients of the AnpIBPs were estimated at approximately 0.1 p.m.^2^/s in the θ direction, indicating that DXB spatially detects picometre-scale motion in live animals (Table S1).

### 3.3 Theta- and chi-directional motion of AnpIBPs in living worms with DXT

**Fig. 3:**
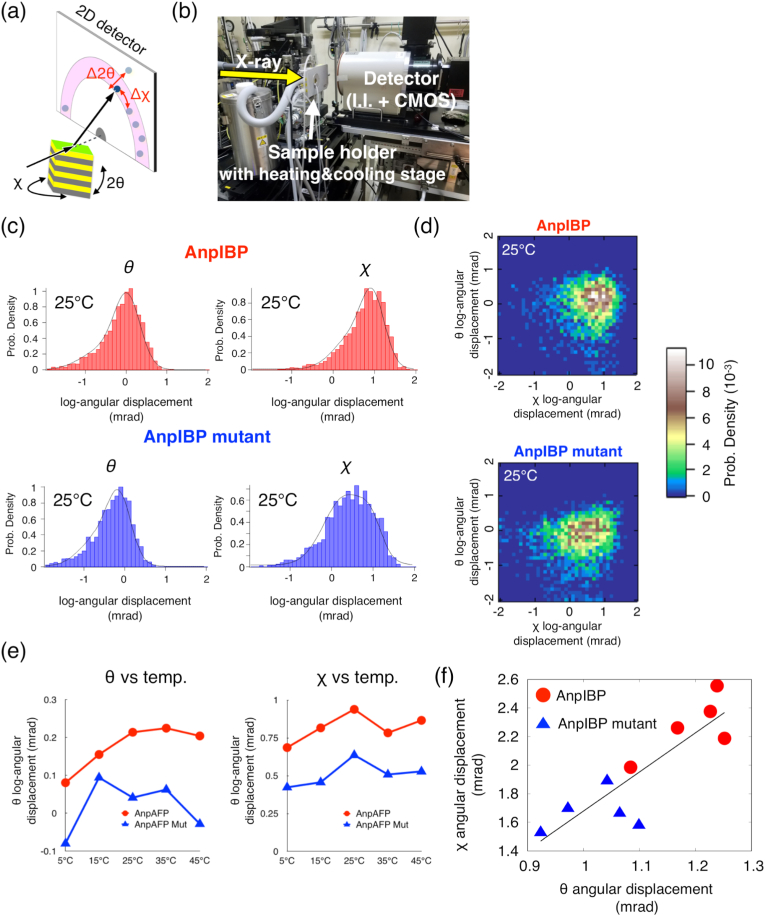
Quantitative analyses of AnpIBP motion in *C. elegans* with diffracted X-ray tracking (DXT). (a) Schematic illustration of DXT measurement. Diffraction spots moving in the θ and χ directions represent the tilting and twisting motions of protein molecules. (b) The DXT setup with pink–beam X-rays in the BL40XU beamline. The diffraction spots were recorded by a CMOS camera with an imaging intensifier for X-ray to light transformation. The sample holders were placed in a heating and cooling chamber. The sample temperature was controlled by a custom-made heating and cooling stage. (c) Probability density of diffraction spot movement for the θ and χ directions at 25 °C. Histograms of all conditions were fitted by a Gaussian distribution. (d) Internal motion probability density maps of AnpIBPs based on Fig. 3c. (e) The graph for θ/χ motion vs. temperature. (f) Angular displacement of θ vs. angular displacement of χ. The red circle indicates wild-type AnpIBP. The blue triangle indicates the AnpIBP mutant. These plots were obtained from the θ and χ motions at 5 °C, 15 °C, 25 °C, 35 °C and 45 °C. (For interpretation of the references to colour in this figure legend, the reader is referred to the Web version of this article.)

The dynamic motion of AnpIBP differed from that of the AnpIBP mutant *in vivo* at each low temperature. The individual diffraction spots probably move in the θ and χ directions. However, knowledge of the orientation of AnpIBP movements is limited from the DXB experiments. DXT tracks the movement of the diffraction spots from the gold nanocrystals by using broadband X-ray and can be separately evaluated for the movements of the θ and χ directions in gold nanocrystals as tilting and twisting [[1], [2], [3], [4], [5],^30^] (Fig. 3a). Here, we performed DXT to observe θ and χ movement in AnpIBPs *in vivo*. To observe the temperature-dependent motion of *C. elegans* AnpIBPs, a heating and cooling stage was equipped with a DXT system (Fig. 3b). The experiments were performed under five temperature conditions (5 °C, 15 °C, 25 °C, 35 °C, and 45 °C). Decreasing the temperature below 0 °C generated ice crystals inside and outside of living worms. The generated ice crystals were detected as high-intensity diffraction spots, and DXT measurements below 0 °C were not performed.

Hundreds of diffraction spots were tracked and evaluated as angular velocities (Figs. S2 and S3). For instance, the peak values of θ and χ angular displacement in AnpIBP at 25 °C appeared to be higher than those in the AnpIBP mutant (Fig. 3c). Internal motion maps for the θ and χ motions also visually showed that the tilting and twisting of AnpIBP were higher than those of the AnpIBP mutant (Fig. 3d). In addition, the χ angular displacement of the AnpIBP mutant showed a broader distribution histogram than those of the wild-type AnpIBP. The peak values of the angular displacement histogram for the θ and χ directions at 5 °C–45 °C are shown as E_θ_ and E_χ_ in Table S2. Next, we analysed the temperature dependency in E_θ_ and E_χ_. The E_θ_ and E_χ_ motions at 5 °C–45 °C in the AnpIBPs seemed to show partially temperature-dependent behaviours (Fig. 3e). The relationship between E_θ_ and E_χ_ in the wild-type AnpIBP and the AnpIBP mutant is shown in Fig. 3f. Interestingly, the θ and χ motions showed that the twisting and tilting motional speeds of AnpIBPs measured above 5 °C were consistently higher than those of the defective AnpIBP mutant (Fig. 3e and f). Based on these findings, we speculate that the faster motions of AnpIBP increase the probability of encountering ice crystals and thus binding to those ice crystals. Importantly, these results demonstrate that the milliradian rotational motion of individual proteins can be quantitatively monitored at the single molecular level in intact animals using DXT.

## 4 Discussion

In this study, we applied the DXB and DXT techniques to observe individual membrane proteins in living *C. elegans*. The rotational motion of transmembrane CD4 and AnpIBPs was observed with picometre precision by DXB. The rotational diffusion coefficients of the AnpIBPs were estimated at approximately 0.1 p.m.^2^/s in the θ direction. Interestingly, the rotational motions of the wild-type AnpIBP were significantly inhibited at −10 °C, whereas those of the mutant AnpIBP did not decrease (Fig. 2d). The wild-type AnpIBP binds to ice crystals, probably decreasing its rotational motion by forming the AnpIBP-ice complex (Fig. 2f). On the other hand, the defective AnpIBP mutant did not bind to ice crystals, so the rotational motion did not show any change. The rotational diffusion includes the tilting and twisting motions of AnpIBPs, suggesting that the rotational diffusion of AnpIBPs directly affected its ice-binding state. These movements of AnpIBPs are consistent with a previous ice docking simulation [16,17].

In this paper, the twisting and tilting motions of AnpIBPs were assigned precisions of approximately 0.4–1.0 mrad and 0.9–2.6 mrad, respectively. We found that the twisting and tilting motional speeds of AnpIBPs measured above 5 °C were consistently higher than those of the defective AnpIBP mutant. Several computational simulations appear to show that IBPs rotate in the χ direction and then bind to ice crystals [16,17]. These fast motions may be effective in orienting the ice-binding site on IBP to a crystal plane of ice, but there is no evidence to support this speculation. Further observations will need to be made to understand this phenomenon by using several mutant IBPs, other IBPs, and in vitro experiments. In addition, we believe that computational simulations are useful to assess this interesting IBP fast motion at above-freezing temperatures, and we plan to perform them in the future.

DXT with a broadband X-ray can obtain the tilting and twisting motions. These motions are related to the ice-binding ability of IBPs. However, DXT measurements below 0 °C were not performed because the generated ice crystals were detected as high-intensity diffraction spots. DXT will provide insight into the IBP-ice interaction at subzero temperatures. The tilting motion probably decreases if AnpIBP-ice complexes are generated at subzero temperatures. The twisting motion also decreases when the protein is bound to ice. Although the twisting motion might have a key role in seeking ice crystals to bind to, it was not clear whether the θ and χ movements of AnpIBPs *in vivo* represent tilting and twisting, respectively. The gold nanocrystal markers attached to CD4 on the intestinal cell membrane might be oriented in various directions. Although several improvements will be required in the labelling and measurement methods, we believe that DXT can be used to evaluate the relationship between tilting-twisting motions and ice-binding ability.

Computational simulations of IBPs have been performed to understand IBP-ice interactions. Generally, IBP simulations have been performed by assuming an equilibrium state at the ice-water interface. Thus, knowledge regarding how IBPs freely interacts with ice crystals in an actively growing state is limited. Recently, Kuiper et al. reported detailed insight into molecular processes during ice crystal inhibition and modification. When IBP is stably bound to the ice-water interface, the melting temperature of the ice decreases. Thereby, the ice-crystal growth surrounding IBP also dramatically decreases. The molecular dynamics simulations suggest that the motion of the IBP mutant increases at subzero temperatures. The IBP mutant is unable to bind to the ice and might diffuse ahead of the growing ice front. These simulations were performed by using IBPs from the spruce budworm *Choristoneura fumiferana* [16] and from the *Tenebrio molitor* [17]. In a crowded environment *in vivo*, the behaviour of IBP is very interesting due to its ice-binding ability. Our paper is the first report to observe the dynamic behaviours of IBPs *in vivo*. A strategy that combines DXB/DXT data and dynamical simulations may be extended to understand IBP dynamics involved in the relationship between structure and function.

Interestingly, diffractions of ice crystals were also observed under frozen conditions of *C. elegans*. The ice shape can be analysed from the 2θ diagram, and their size can also be calculated by the Scherrer equation [31]. In addition, DXT and DXB are capable of temporally monitoring their dynamic motions and ice crystal growth. Lethal ice formation should be minimally inhibited in a solidification process for cryopreservation [32]. We believe that observations of ice crystal formation *in vivo* are useful to establish optimal cooling and warming methods for cryopreservation in animals.

Finally, we developed the DXB and DXB *in vivo* techniques described here to observe molecular motion in live animals. The deployment of our metrology techniques has now been expanded from protein and cell to a live animal. The application of DXB and DXT will shed light on the molecular basis of complex biological phenomena in biochemistry, biophysics, pharmacology, pathology, and neuroscience with atomic-scale precision.

## Author contributions

M.K. and Y.C.S. designed the experiments. M.K., Y.D., Y.S., M.D., K.A., H.S. and K. M. performed the experiments. M.K., Y.D., Y.Y., T.A., and R.O. analysed the data. M.K., S.T. and Y.C.S. wrote the manuscript. All authors discussed the results and contributed to the final manuscript.

## Declaration of competing interest

The authors declare that they have no known competing financial interests or personal relationships that could have appeared to influence the work reported in this paper.

## Data Availability

Data will be made available on request.
