# Supplementary material for: Dynamic motions of ice-binding proteins in living Caenorhabditis elegans using diffracted X-ray blinking and tracking
Source: Biochem Biophys Rep. 2022 Feb 3;29:101224. doi: 10.1016/j.bbrep.2022.101224 (PMC8819013; doi:10.1016/j.bbrep.2022.101224)
Supplement: Multimedia component 1 [file mmc1.docx]

**Supplemental Materials**

**Fig. S1.** **Distribution of the ACF decay constant.**

Histograms of wild-type AnpIBP (a) and a defective mutant AnpIBP T156Y (b) are represented as boxplot graphs in Fig. 2e. DXB experiments were performed at -10 °C, -5 °C, 0 °C, 5 °C, 10 °C, and 20 °C.

**Fig. S2. Trajectory of diffraction spot movement.**

These panels represent the actual motion of the diffraction spots in the θ and χ directions. DXT experiments were performed at 5 °C, 15 °C, 25 °C, 35 °C and 45 °C. The trajectories of the AnpIBP and AnpIBP mutants were analysed based the θ-χ coordinates. The number of trajectories is presented at the centre of each panel.

**Fig. S3. Distribution of the absolute angular displacement in the θ and χ directions.**

Angular displacement distribution of the AnpIBP- or AnpIBP mutant-CD4 complex with gold nanocrystals on intestinal cells at 5 °C, 15 °C, 25 °C, 35 °C and 45 °C. The common logarithm of the angular velocity was used to map the probability density. Histograms were fitted by a Gaussian distribution.

**Fig. S4. Internal motion maps of IBPs.**

Internal motion probability density maps of AnpIBP (a) and a defective AnpIBP mutant (b) at 5 °C, 15 °C, 25 °C, 35 °C and 45 °C. The logarithm of the absolute displacements in Fig. S3 was used to draw the 2D histogram. The horizontal and vertical axes are the θ (tilting) and χ (twisting) directions, respectively.

**Table S1. Mean ACF decay constants and rotational diffusion coefficients.**

|  | Temp. (℃) | DXB | |
| --- | --- | --- | --- |
|  |  | Median value of ACF decay constant (sec^-1^) | Rotational diffusion coefficient  (pm^2^/sec) |
| AnpIBP | 20 | 0.0349 | 0.1194 |
|  | 10 | 0.0304 | 0.1040 |
|  | 5 | 0.0341 | 0.1167 |
|  | 0 | 0.0307 | 0.1051 |
|  | -5 | 0.0344 | 0.1177 |
|  | -10 | 0.0283 | 0.0969 |
| AnpIBP  Mutant | 20 | 0.0278 | 0.0951 |
|  | 10 | 0.0304 | 0.1040 |
|  | 5 | 0.0298 | 0.1020 |
|  | 0 | 0.0279 | 0.0955 |
|  | -5 | 0.0339 | 0.1160 |
|  | -10 | 0.0362 | 0.1239 |

**Table S2. Peak values E_θ_ and E_χ_ in angular displacement for θ and χ.**

|  | Temp. (℃) | DXT | |
| --- | --- | --- | --- |
|  |  | E*_θ_* (mrad) | E*_χ_* (mrad) |
| AnpIBP | 45 | 1.0839 | 1.9853 |
|  | 35 | 1.1678 | 2.2612 |
|  | 25 | 1.2385 | 2.5557 |
|  | 15 | 1.2519 | 2.1880 |
|  | 5 | 1.2265 | 2.3766 |
| AnpIBP  mutant | 45 | 0.9233 | 1.5266 |
|  | 35 | 1.0988 | 1.5785 |
|  | 25 | 1.0420 | 1.8892 |
|  | 15 | 1.0644 | 1.6619 |
|  | 5 | 0.9720 | 1.6956 |
